# A rare case of post-transplant lymphoproliferative disorder (Hodgkins lymphoma) post autologous stem cell transplantation: A case report and review of literature

**DOI:** 10.1016/j.amsu.2022.104738

**Published:** 2022-09-22

**Authors:** Subaina Naeem Khalid, Najah Zari Amir, Zeest Ali Khan, Amin Moazzam Khan, Rehab Naeem Khalid, Muhammad Hamza Ali, Ibad ur-Rehman, Khabab Abbasher Hussien Mohamed Ahmed, Irfan Ullah

**Affiliations:** a Shifa College of Medicine, Shifa Tameer e Millat University, Islamabad, Pakistan; b Agha Khan University, Karachi, Pakistan; c Shifa International Hospital, Islamaba, Pakistan; d Faculty of Medicine, University of Khartoum, Khartoum, Sudan; e Department of Internal Medicine, Kabir Medical College, Gandhara University, Peshawar, Pakistan; f Institute of Public Health & Social Sciences (IPH&SS), Khyber Medical University, Peshawar, Pakistan

**Keywords:** Case report, Bone marrow transplant, Lymphoma, Chemotherapy

## Abstract

**Introduction:**

Post-transplant lymphoproliferative disorders(PTLD) include a mix of rare yet life endangering complications.

**Case presentation and conclusion:**

Here, we report a case of a 63-year-old man who was the victim of post-transplant lymphoproliferative disorder (Hodgkin's lymphoma). The patient was initially diagnosed with multiple myeloma, for which chemotherapy and the autologous stem cell transplant was carried out. Post transplant patient was stable but on a follow up visit 6 months after the transplant he presented with generalized lymphadenopathy.

His subsequent workup was done including a biopsy of cervical lymph node, which revealed Classical Hodgkin's lymphoma (post-transplant lymphoproliferative disorder (PTLD)) of mixed cellularity type.

The patient was started on chemotherapy and received a total of 4 cycles of Chemotherapy (ABVD) before his condition started deteriorating as chemotherapy was poorly tolerated leading to Bleomycin toxicity. The patient regrettably passed away due to an NSTEMI.

## Highlights

• Post-transplant lymphoproliferative disorders (PTLD) are a heterogeneous group of major life-threatening complications associated with hematopoietic stem cell transplants (HSCT), bone marrow transplants, and solid organ transplants (SOT) with extremely diversified presentations, classical Hodgkin Lymphoma (cHL)variant of PTLD being the rarest. To the best of our knowledge, this is the first reported case of cHL variant PTLD in a patient of autologous stem cell transplant for multiple myeloma.
•We present a case of Multiple Myeloma that was successfully treated with 3 cycles of bortezomib-based triple therapy (bortezomib, Thalidomide, and dexamethasone) following which autologous stem cell transplantation was performed. 3 years after successful treatment the patient was diagnosed with Classical Hodgkin's lymphoma (post-transplant lymphoproliferative disorder (PTLD)) of mixed cellularity type.
•He was treated with ABVD chemotherapy regimen (Doxorubicin, Vinblastine, Dacarbazine, and Bleomycin), and poorly tolerated the fourth cycle of chemotherapy following which he developed Bleomycin toxicity. The patient, unfortunately, passed away from a sudden episode of Non-ST-Elevation Myocardial Infarction (NSTEMI).

## 1 Introduction

Post-transplant lymphoproliferative disorders (PTLD) are a heterogeneous group of major life-threatening complications of hematopoietic stem cell transplants (HSCT), bone marrow transplants, and solid organ transplants (SOT) [1]. Given that the clinical presentations of PTLD can be extremely diversified, ranging from benign to severely malignant, the World Health Organization has classified PTLD into six categories as follows: classical Hodgkin lymphoma PTLD, plasmacytic hyperplasia PTLD, polymorphic PTLD, monomorphic PTLD (B- and T-/natural killer-cell types), infectious mononucleosis PTLD and florid follicular hyperplasia PTLD [2]. In 50–80% of PTLD cases Epstein- Barr virus (EBV) reactivation in patients on immunosuppressive therapy is the major culprit implicated in disease onset, although EBV negative cases are seen as well [3]. The factors determining a higher incidence of PTLD include the type of organ transplanted, age and race of recipient, the potency of immunosuppressive drugs administered as well as genetic polymorphisms [4,5]. In this case report we present a case of the Classical Hodgkin Lymphoma (cHL) variant of PTLD, which is EBV positive, post autologous stem cell transplant (ASCT) carried out for the treatment of multiple myeloma. This case report adheres to the SCARE criteria and is being reported in accordance with the guidelines [6] To the best of our knowledge, this is the first case of cHL variant PTLD in a patient of autologous stem cell transplant for multiple myeloma.

## 2 Case presentation

A 63-year-old male presented to the out-patient clinic with generalized weakness. He had a BMI of 19.1kg/m2 and was a known case of insulin-controlled diabetes for the past 4 years. On laboratory evaluation, the patient's complete blood count revealed anemia. Bone marrow aspiration showed binucleate plasma cells, and serum protein electrophoresis revealed monoclonal gammopathy. Furthermore, his Beta-2 microglobulin level was 4.9mg/dL in the gamma region. The immune-electrophoresis confirmed IgG kappa and a skeletal survey showed lytic lesions in the skull, which confirmed the diagnosis of multiple myeloma. The patient underwent 3 cycles of bortezomib-based triple therapy (bortezomib, Thalidomide, and dexamethasone) after which a remission assessment was carried out that demonstrated consolidation. However, the patient had developed a fair amount of neuropathy. The protein electrophoresis was then repeated which did not display any evidence of paraproteinemia. Moreover, his Beta-2 microglobulin levels reduced to 4.0 and he was admitted for cyclo-G mobilization. He was started on Injection Granulocyte-Colony Stimulating Factor at day-7 post cyclophosphamide. After that, peripheral blood stem cell (PBSC) harvest was collected in 2 sessions which was used for autologous stem cell transplant. Autologous stem cell transplant was carried out using high dose Melphalan (200mg/m2 on day −1 after completion of stem cell harvest) as conditioning chemotherapy. He was started on antibiotics (moxifloxacin) once daily, antifungal (fluconazole), and antiviral (acyclovir) prophylactically. He was also administered mononuclear cells (MNC) and CD34 at a dose of 2.58x 10^8 kg and 11.75 x 10^6 kg respectively.

**Fig. 1:**
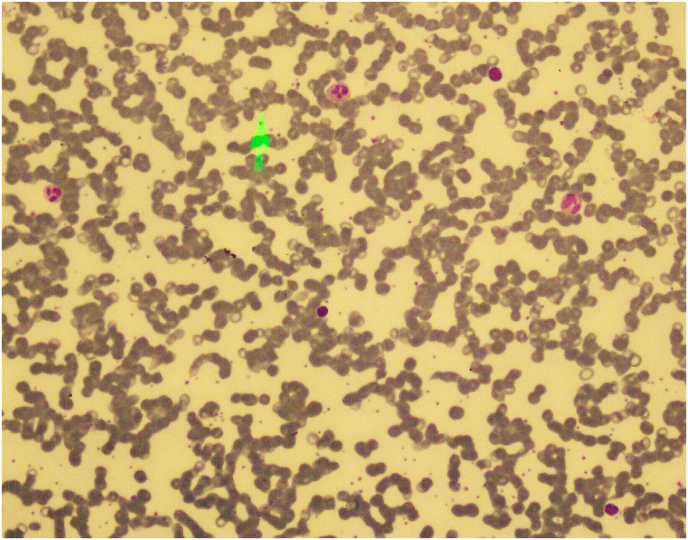

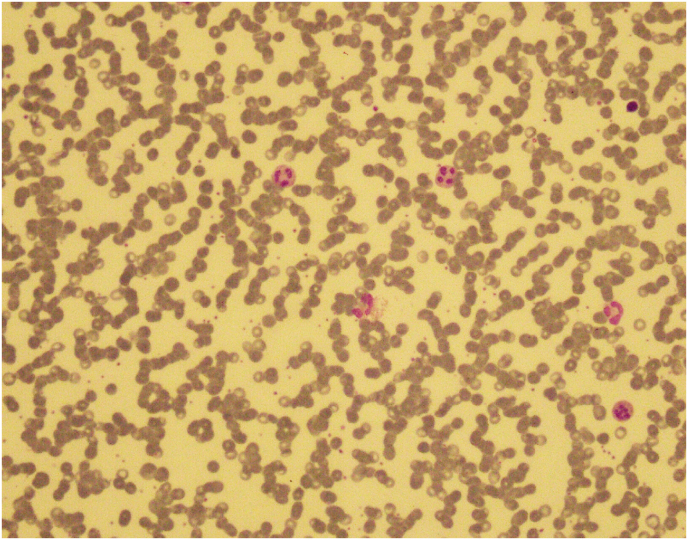
(a,b): Peripheral blood film showing red blood cell Rouleaux formation.

**Fig. 2:**
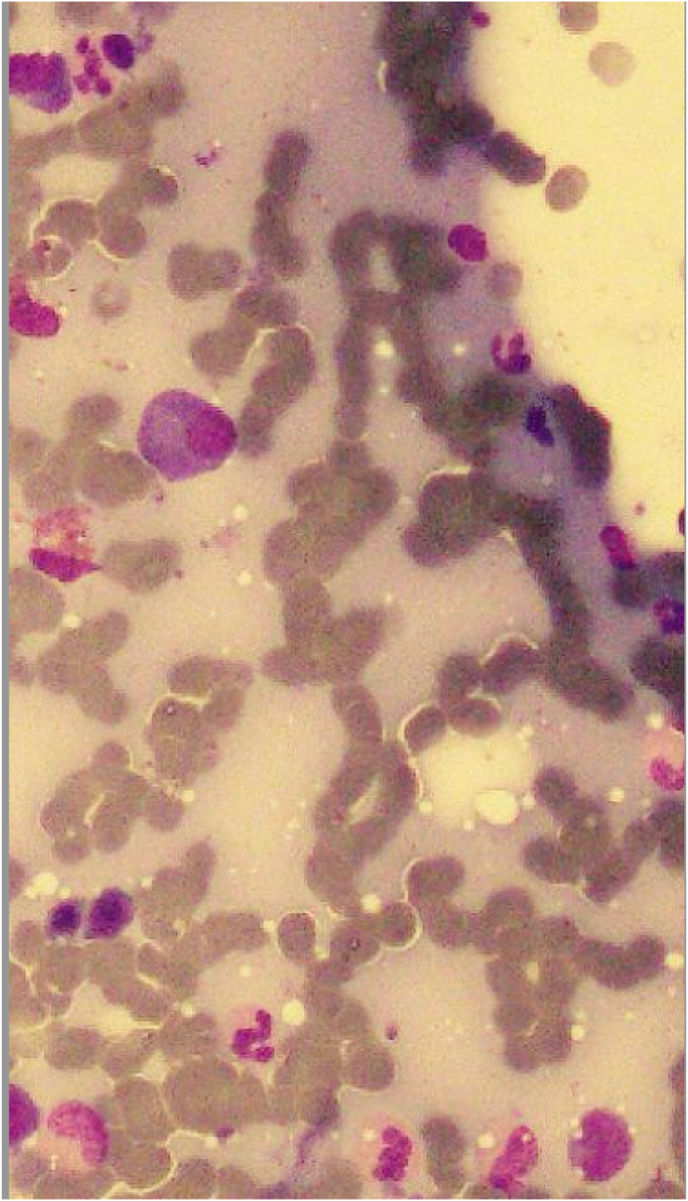
Bone marrow aspirate showing plasma cells.

**Fig. 3:**
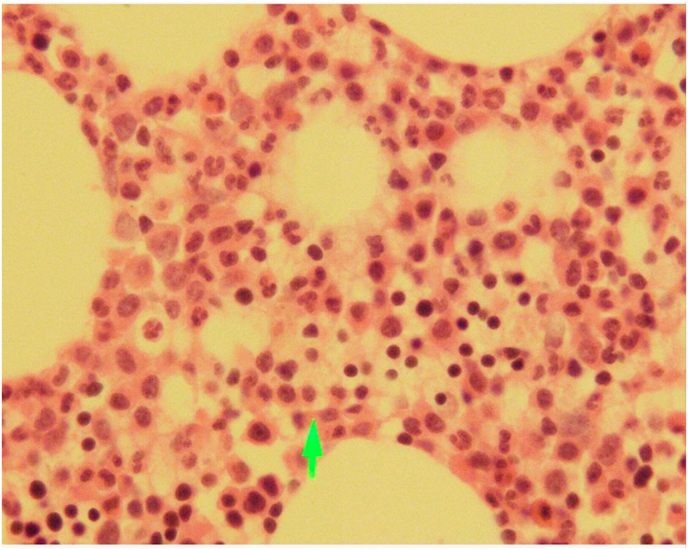
Trephine biopsy showing plasma cells.

During transplant, the patient developed grade-3 mucositis and cryptosporidiosis. On day 6, the patient developed febrile neutropenia for which IV Tazobactam-Piperacillin, Amikacin, and Amphotericin-B were given. Blood C/S was done which showed no growth of bacteria. Furthermore, the patient achieved neutrophil engraftment on day 10, and platelet engraftment on day 12 post-transplant. He was discharged on the 16th day after the transplant with normal blood counts and was advised to stay on antiviral and *anti*-PCP prophylaxis for 6 months after the procedure. 6 months post-transplant, the patient fulfilled disease reassessment criteria. He maintained a good quality of life and was able to resume his regular activities as well as his professional responsibilities for the following three years after the transplant. In January 2019, following a tooth extraction, the patient presented to the OPD with generalized lymphadenopathy, fever, persistent hiccups along with bi-cytopenia. Upon performing a Contrast-enhanced CT scan of the chest, abdomen, and pelvis; prominent right hilar lymph nodes and para esophageal lymph nodes measuring 10mm and 11mm respectively were seen. Excisional Biopsy of cervical lymph node was performed, which revealed Classical Hodgkin's lymphoma (post-transplant lymphoproliferative disorder (PTLD)) of mixed cellularity type (refer to Fig. 1, Fig. 2, Fig. 3). His EBV-PCR showed 35410 copies/ml and CD30 immune-histology was positive. He subsequently started on ABVD chemo-therapy (as elaborated below). Each 4-week cycle consisted of the following drug regimen on day 1 and then on day 15:

Day1 Doxorubicin 25mg/m2 IV Bolus.

Day1 Vinblastine 6mg/m2 IV infusion in 50mL NaCl 0.9% over 10mins.

Day1 Dacarbazine 375mg/m2 IV Infusion in 250–500 ml NaCl 0.9% over 1–2 hours.

Day1 Bleomycin 10,000 units/m2 in 100ml NaCl 0.9% IV infusion over > 1hour.

He tolerated the chemotherapy well and started improving after the second cycle.

However, following the 4th cycle of ABVD, he started complaining of shortness of breath. A High-resolution CT scan (HRCT) of the chest was done, revealing bronchiectatic changes in bilateral basal segments and middle lobes of the lungs. The interstitial lung disease was thought to be due to bleomycin toxicity. HRCT also revealed prominent lymph nodes in the pre-vascular region, AP window, and right paratracheal region with the largest one measuring 7.3mm. The patient's deteriorating condition required supportive oxygen therapy and antibiotics. Unfortunately, he had a sudden episode of Non-ST-Elevation Myocardial Infarction (NSTEMI) which ultimately proved to be the cause of his death. . This case report adheres to the SCARE criteria.

## 3 Discussion

Classical Hodgkin Lymphoma PTLD has the lowest incidence amongst all six subcategories of PTLD. PTLD after hematopoietic stem cell transplant is in itself rare ranging from 1.16% to 8% depending upon the presence of risk factors and the incidence of cHL in cases of PTLD is reported to be 1.8%–3.4% in two separate series. Therefore the rarity of cHL post ASCT can be appreciated [7]. There is a stringent criterion to be fulfilled to diagnose cHL which was duly fulfilled in our patient's case with excisional biopsy of lymph nodes showing a picture of cHL of mixed cellularity subtype and immunophenotyping showing CD30 positivity. It is important for a correct diagnosis to be made as Reed-Sternberg-like cells are seen in non-destructive PTLDs which would change the management strategy [2]. Greater than 80% of PTLD cases after HSCT are diagnosed in the first year after transplantation but our patient was diagnosed 3 years post-transplantation.

PTLD after being recognized in 1968 and officially being dubbed PTLD in 1984 has been given due diligence due to its potential deadliness. Till 1999 attributable mortality associated with post-HSCT PTLD was 84.6% [8,9]. Introduction of several methods of prophylaxis and monitoring has led to considerable improvement in mortality, but it is still notably high-which is one-third of diagnosed cases. According to the sixth European Conference on Infections in Leukemia (ECIL-6) guidelines, EBV DNA monitoring using PCR should start in the first month of HSCT being carried out and should be done every week until after four months of HSCT. Tailoring such monastic surveillance to the individual is important as those who have increasing levels of EBV DNA should have an increased frequency of monitoring. Our case shows that further exploration of the duration for which monitoring should be carried out is warranted given its rare late presentation after 3 years [10]. Interventions to prevent rising levels of EBV DNA to essentially halt progression to PTLD would need concrete cut-off values of EBV DNA, but that is not the case as no set range has been standardized. Furthermore, there is no conclusive evidence whether EBV DNA obtained from mononuclear cells, blood or plasma is the most superlative. This, therefore, points to centers standardizing their own EBV DNA ranges by PCR, and the rate of increment of EBV DNA as a measure of deciding when to intervene is indispensable [11].

The management strategies prior to the development of EBV positive PTLD are twofold. The first is aimed at keeping the viral load of EBV in check in any patient who is seropositive for EBV; these patients would have no symptoms and the second strategy is to prevent EBV disease with clinical manifestations in a patient with increasing viral loads. A few of these strategies can be carried out by the administration of rituximab, EBV-specific cytotoxic T cells, and reduction of maintenance immunosuppression i.e., at least twenty percent of daily dosage barring low-dose corticosteroids [10,11]. The usage of post-transplant anti-viral drugs has not been shown to have therapeutic efficacy for keeping EBV viral loads at bay and our patient had been on antivirals and *anti*-Pneumocystis pneumonia prophylaxis for 6 months, but the treatment of latent EBV with antivirals has shown to be futile as B cells with latent infection do not express the EBV thymidine kinase enzyme transcript or protein [10,12,13].

Once the diagnosis of cHL was established, the patient started on the standard ABVD (Doxorubicin, Bleomycin, Vinblastine, Dacarbazine) chemotherapy regimen. With the usage of Bleomycin in the elderly patient, there is an increased risk of pulmonary toxicity,not only at a higher rate but a higher grade of pulmonary toxicity has also been reported in the elderly who received 4 cycles of ABVD as opposed to 2 cycles of ABVD or AVD [14]. This had to be weighed against poorer tumor control when eliminating Bleomycin from the regimen [15]. The increased likelihood of relapse then led to the decision of 4 cycles of ABVD in our patient which was unfortunately poorly tolerated leading to Bleomycin toxicity. The patient regrettably passed away due to an NSTEMI.

## Ethical approval

We further confirm that any aspect of the work covered in this manuscript that has involved human patients has been conducted with the ethical approval of all relevant bodies and that such approvals are acknowledged within the manuscript. IRB approval was obtained. Written consent to publish potentially identifying information, such as details or the case and photographs, was obtained from the patient(s) or their legal guardian(s).

## Sources of funding

This research was funded by the authors themselves.

## Author contribution

**SNK**,NZ, ZAK, AMK: conceived the idea, designed the study, drafted the manuscript and gave the final approval. RNK, MHA, IR,IU, AHMA: conducted literature search and created illustrations, revised the manuscript.

## Research registration studies

Name of the registry: NOT APPLICABLE.

Unique Identifying number or registration ID:

Hyperlink to your specific registration (must be publicly accessible and will be checked):

## Guarantor

1.Ibad ur Rehman.

Shifa college of Medicine, Shifa Tameer e Millat University, Islamabad, Pakistan. ibadrehmaan@gmail.com.

## Consent

Written informed consent was obtained from the patient for publication of this case report and accompanying images. A copy of the written consent is available for review by the Editor-in-Chief of this journal on request.

## Provenance and peer review

Not commissioned, externally peer-reviewed.

## Declaration of competing interest

None to declare.
